# Characterizing renal involvement in Hermansky-Pudlak Syndrome in a zebrafish model

**DOI:** 10.1038/s41598-019-54058-5

**Published:** 2019-11-27

**Authors:** H. Schenk, J. Müller-Deile, P. Schroder, P. Bolaños-Palmieri, L. Beverly-Staggs, R. White, J. H. Bräsen, H. Haller, M. Schiffer

**Affiliations:** 1 Department of Medicine/Nephrology, Hannover Medical School, 30625 Hannover, Germany; 2 Mount Desert Island Biological Laboratory, Salisbury Cove, ME 04672 USA; 3 Department of Nephrology and Hypertension, University of Erlangen-Nurnberg, Erlangen, Germany; 4 Institute of Pathology, Nephropathology Unit, Hannover Medical School, Hannover, Germany

**Keywords:** Glomerular diseases, Focal segmental glomerulosclerosis

## Abstract

Hermansky-Pudlak Syndrome (HPS) is a rare disease caused by mutations in the genes coding for various HPS proteins. HPS proteins are part of multi-subunit complexes involved in the biogenesis of organelles from the lysosomal-endosomal-system. In humans, this syndrome is characterized by the presence of albinism, platelet dysfunction and pulmonary fibrosis. The renal component to the disease remains unstudied and untreated in patients with HPS. Here we demonstrate that in humans, HPS proteins have a high renal expression with active transcription of HPS1, 3, 4 and 5 in human podocyte cell culture, suggesting that impaired function of HPS proteins could directly impact renal function. Therefore, we developed a zebrafish model to study the renal involvement of HPS proteins in proteinuric kidney disease. Remarkably, knockdown of HPS genes in zebrafish causes glomerular injury with edema, proteinuria and structural changes of the glomerular filtration barrier. Moreover, reduced expression of HPS proteins in zebrafish recapitulates other important disease hallmarks, like hypopigmentation and accumulation of intracellular debris characteristic of lysosomal disorders. In conclusion, we present a valid zebrafish model that highlights the previously underestimated relevance of renal disease in HPS. This draws attention to the therapeutic options available to manage this component of the syndrome.

## Introduction

Hermansky-Pudlak Syndrome (HPS) is a rare autosomal recessive genetic disorder characterized by the presence of oculocutaneous albinism, platelet storage pool deficiency that causes a haemorrhagic diathesis as well as lysosomal accumulation of ceroid lipofuscin which results in pulmonary fibrosis amongst others^1–3^. In 1959, Hermansky and Pudlak initially described a case of a woman with albinism that presented, aside from the primary HPS characteristics including coagulopathy, with the presence of pigmented reticuloendothelial cells in the kidney as well as an impaired renal function^1^. It has been recently reported that proteinuric kidney disease may be associated with the presence of HPS^4^. The accumulation of renal lysosomal ceroid lipofuscin marks the involvement of the kidney in HPS, however, the understanding of the underlying pathophysiological mechanism is very limited. In a case series comprising 49 patients suffering from HPS, nearly 20% had been diagnosed with chronic kidney disease^3^. Unfortunately, neither histologic analysis of renal tissue nor the quantification of proteinuria were collected to further determine the pathogenesis of the high prevalence of the renal insufficiency^3^. Gordillo *et al*. documented the first case of a pediatric patient suffering from HPS type I with biopsy-proven focal segmental glomerulosclerosis (FSGS) and prevalent chronic kidney disease stage II, accompanied by proteinuria of 30 mg/dl and hypoalbuminemia without hematuria^4^. Furthermore, electron microscopy analysis revealed the presence of podocyte foot process effacement, indicative of a podocytopathy^4^. Given the current stage of research, it is therefore unknown whether proteinuric kidney disease, for example FSGS, appears frequently in HPS types and could therefore explain the high percentage of kidney disease cases in HPS. In previous studies, lysosomal dysregulation has been associated directly with podocytopathies such as FSGS, therefore, HPS may serve as a model disease for lysosome associated podocythopathies^5–7^. The vast majority of all HPS cases can be attributed to pathogenic variants of the HPS1 gene, especially in patients of Puerto Rican descent. Other pathogenic polymorphisms in HPS3, HPS4 and HPS5 appear with a proportion of around 10% each in non-Puerto Rican patients, while disease associated variants in HPS6 are less frequent and are mostly limited to small populations associated with limited geographical distribution. The HPS genes encode for proteins that function as components of BLOCs (Biogenesis of Lysosome-related Organelles Complexes). BLOC3 is present as a heterodimer consisting of HPS1 and HPS4 supposedly involved in endosome and lysosome biogenesis and transport. A dysfunction of BLOC3 resulting from mutations in its components HPS1 and 4 leads to the most severe clinical manifestations of the disease often associated with pulmonary fibrosis and colitis^3^. HPS3, 5 and 6 form a heterotrimer, the BLOC2, which functions as a modifier of lysosome excretion, binds microtubuli and regulates clathrin mediated endocytosis^8^.

In zebrafish, orthologues of HPS1, -3, -4, and -5 are present. Using our zebrafish (*Danio rerio*) pronephros model, we are able to identify proteins which, when their regular expression is disrupted, might be associated with glomerular diseases that share podocyte damage as a hallmark. In order to identify these podocytopathies, we developed an animal model to evaluate disease mechanisms for the incidence of proteinuric kidney disease in HPS using the transgenic zebrafish-line Tg(*l-fabp*:eGFP-DBP) as previously described^9^. This zebrafish line expresses a circulatory protein of similar size to human albumin, namely vitamin-D-binding protein tagged with enhanced GFP (eGFP-DBP) under the control of the fabp-promoter^10^. Analog to albumin, eGFP-DBP cannot cross the glomerular filtration barrier under physiologic conditions partly due to its size of approximately 78 kDa. In this model, the loss of the fusion protein from the circulation of each zebrafish may indicate a dysfunction of the glomerular filtration barrier (GFB). The identification of protein loss is furthermore correlated with the presence of pericardial effusion and yolk sac edema which has been associated with the loss of oncotic pressure^11,12^. To determine whether these hallmarks of proteinuric kidney diseases result from glomerular defects, we used transmission electron microscopy (TEM) which enabled us to further determine the presence of glomerular pathologies.

To induce a specific knockdown (KD) of the genes involved in HPS etiology we performed morpholino (MO-) injection at the zygote stage and determined the gene specific kidney related function *in vivo*.

Given the fact that the type of kidney disease and the pathophysiological mechanisms responsible for the renal involvement in HPS remain elusive, and that therapeutic options in HPS do not include any medication which targets renal disease, we carried out this study to decipher this component of HPS.

## Results

### mRNA of HPS1, HPS3, HPS4 and HPS5 is expressed in human podocytes and glomerular endothelial cells but not in glomerular mesangial cells

**Figure 1:**
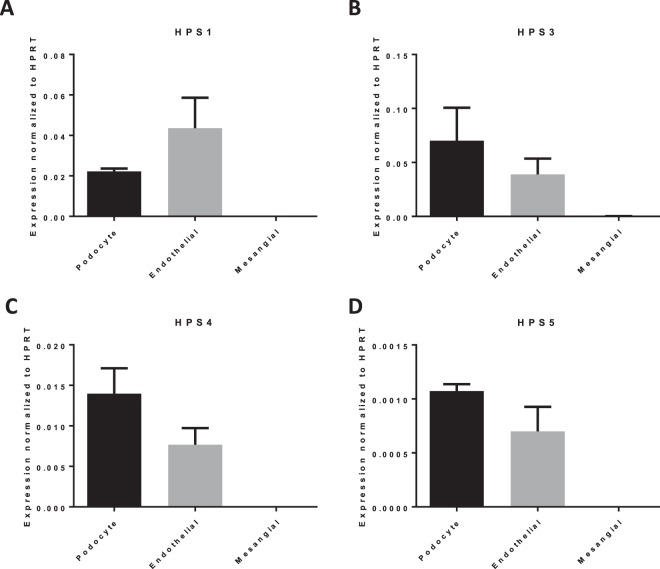
Expression of mRNA of HPS subtypes in human renal cell lines. Quantitative PCR (q-PCR) reveals mRNA levels of HPS1 (**A**), HPS3 (**B**), HPS4 (**C**) and HPS5 (**D**) normalized to the housekeeping gene hypoxanthin phosphoribosyl transferase (HPRT) in cultured human podocytes, human glomerular endothelial cells and human mesangial cells. HPS1 mRNA expression can be detected in podocytes and at the highest level in glomerular endothelial cells, while no expression is present in mesangial cells. Analog to HPS1, HPS3 mRNA is expressed in podocytes and endothelial cells, mesangial cell expression cannot be detected. The predominant expression is present in podocytes. HPS4 mRNA is furthermore detected predominately in podocytes and to a lesser extent in endothelial cells, mesangial cell expression is not present. HPS5 mRNA shows its highest expression in podocytes, it can be detected in endothelial cells, but not in mesangial cells. Error bars represent the mean normalized expression ± SEM between two independent experiments (n = 2).

We examined the mRNA expression of the above named HPS genes in cell culture lines of human podocytes, glomerular microvasculature endothelial cells as well as glomerular mesangial cells and normalized the expression to human HPRT (Fig. 1). We detected that HPS1 mRNA expression in human cultured podocytes is lower compared to human glomerular microvasculature endothelial cells (Fig. 1A). In contrast, HPS1 mRNA was not detected in human glomerular mesangial cells (Fig. 1A). The expression of HPS3 mRNA was primarily detected in human podocytes, in human endothelial cells, however, HPS3 expression is lower (Fig. 1B). Mesangial cell expression HPS3 mRNA was not detectable (Fig. 1B). A similar mRNA expression distribution was furthermore present with HPS4 (Fig. 1C). HPS4 mRNA was mostly expressed in human podocytes, while endothelial cell expression was markedly lower while no mesangial cell expression was present (Fig. 1C). HPS5 mRNA expression was also present in podocytes and endothelial cells, however, it was markedly lower in comparison with HPS1, HPS3 and HPS4 (Fig. 1D). Parallel to these, mesangial cell expression of HPS5 mRNA was also not detected (Fig. 1D).

### Immunohistochemistry of human kidney samples demonstrates that HPS genes are predominately expressed in the tubuli, glomerular expression varies markedly between types

**Figure 2:**
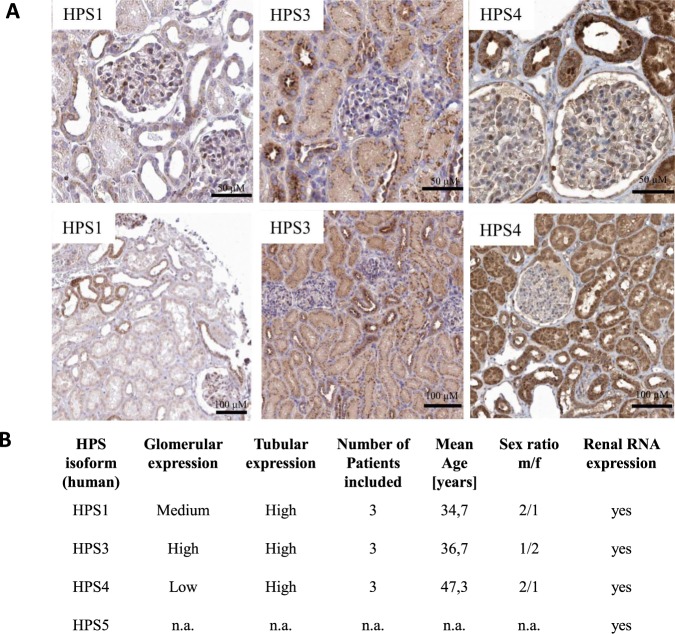
Cellular expression of HPS proteins was analyzed by immunohistochemistry in human kidney sections. (**A**) Localization of HPS 1 expression in glomeruli was present at a medium level, while high expression of HPS1 was prevalent in tubuli (https://www.proteinatlas.org/ENSG00000107521-HPS1/tissue/kidney). Expression of HPS3 in glomeruli was high, while a dominant expression in tubuli was detected (https://www.proteinatlas.org/ENSG00000163755-HPS3/tissue/kidney). HPS4 showed only minor expression in glomeruli, however, strong expression in tubuli was present (https://www.proteinatlas.org/ENSG00000100099-HPS4/tissue/kidney). No data on HPS5 protein expression can be accessed through Protein Atlas (https://www.proteinatlas.org/ENSG00000110756-HPS5/tissue/kidney). All described data were obtained using Protein Atlas Version 18. (**B**) Data on presence of HPS protein subtypes in human kidney sections. Data on HPS expression pattern is pooled from three kidney sections each. Furthermore, the number of patients included, the mean age, sex ratio and the renal RNA expression are stated.

Based on RNA seq data derived from biopsy samples of human kidneys (n = 3) available in the human protein atlas website (www.proteinatals.com), we can confirm that, congruent with our own cell culture data, all HPS forms are actively transcribed in the kidney. Similarly, immunohistochemistry analysis from these sections shows that most HPS forms are ubiquitously expressed in renal cells, with especially strong signal in the tubuli (Fig. 2A). According to the available data in the human protein atlas, the primary localization of HPS1 is tubular, however, its expression can also be detected in glomerular cells. HPS3 is expressed at a strong level in both tubular and glomerular cells. HPS4 has the most prevalent tubular expression of the studied genes, while its glomerular expression is markedly lower compared to HPS1 and HPS3. For all studied HPS genes, renal RNA expression was detected (Fig. 2B). Unfortunately, no data on the HPS5 protein expression was available in the human protein atlas.

### Analysis of human and zebrafish HPS proteins reveals a high resemblance between orthologues of HPS genes

**Figure 3:**
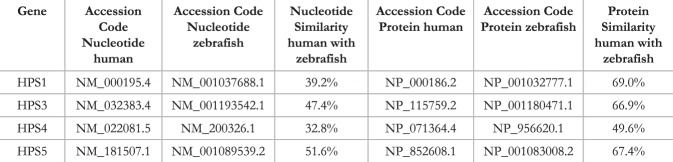
Comparison of the HPS nucleotide and proteins in zebrafish and human orthologues. The nucleotide and protein sequence similarity between human and zebrafish HPS are stated. Accession codes of protein and nucleotide sequences from both organisms are shown. Table was generated with the help of NCBI (https://www.ncbi.nlm.nih.gov/gene) and EMBOSS tools (https://www.ebi.ac.uk/Tools/psa/emboss_stretcher and https://www.ebi.ac.uk/Tools/psa/emboss_stretcher/nucleotide.html).

We examined the similarity between the sequences of each human HPS gene and the corresponding zebrafish orthologue at both the nucleotide and protein level by the use NCBI gene (https://www.ncbi.nlm.nih.gov/gene) and EMBOSS tools (https://www.ebi.ac.uk/Tools/psa/emboss_stretcher and https://www.ebi.ac.uk/Tools/psa/emboss_stretcher/nucleotide.html). Nucleotide sequence similarity is highest between HPS5 genes, slightly above 50%; whereas the HPS4 loci have the lowest similarity, at 32.8%. At the protein level, the similarity is much higher between both organisms varying between 49.6% for HPS4 up to almost 70% for HPS1 (Fig. 3). This level of sequence similarity between zebrafish and humans highlights the importance of this group of proteins within an evolutionary context, suggesting the presence of a conserved biological function. This allows us to further characterize their role in kidney function, as well as the possible pathophysiological consequences of their absence, by using the zebrafish as a model organism.

### Knockdown of HPS1 (HPS^1KD*i*6*e*7^, HPS1^KD ATG^) and -5 (HPS5^KD e12i12^, HPS5^KD ATG^) results in a severe renal phenotype with pericardial effusion, yolk sac edema and protein loss, while HPS3 (HPS^3KD*e*7*i*7^, HPS3^KD ATG^) and -4 (HPS4^KD e8i8^, HPS4 ^KD ATG^) induces, if present at all, only mild pericardial effusion, yolk sac edema and no protein loss in zebrafish larvae

**Figure 4:**
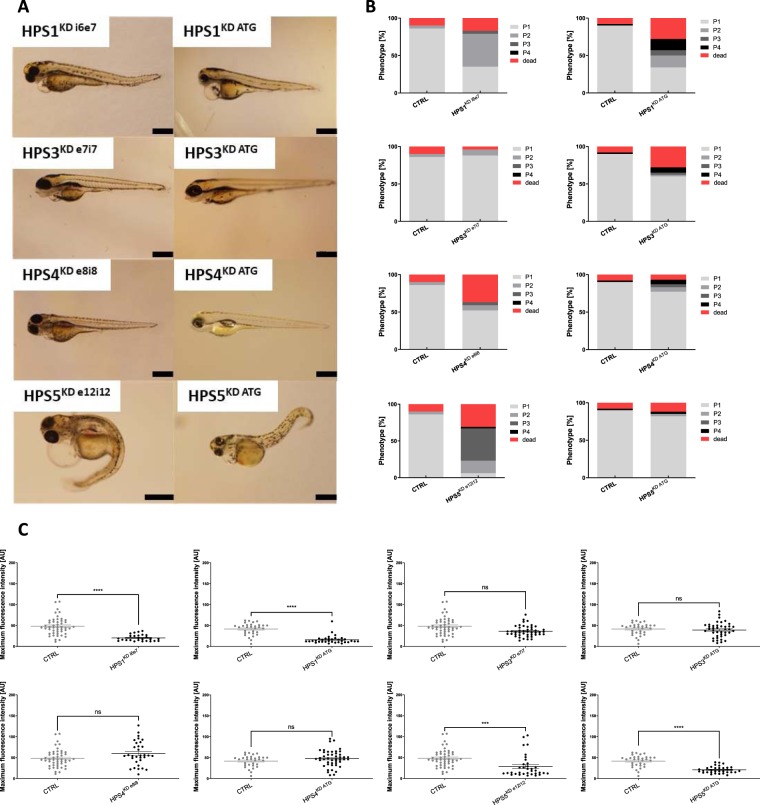
Phenotype evaluation and renal protein loss determination after induction of HPS1^KD^, HPS3^KD^, HPS4^KD^ and HPS5^KD^ in zebrafish. (**A**) The larval phenotype was analyzed at 96 hpf following HPS^KD^ at the 1–2 cell stage. Both HPS1^KD i6e7^ and HPS1^KD ATG^ larvae showed pericardial effusion and yolk sac edema and a slight curvature of the tail. HPS3^KD e7i7^ and similarly HPS3^KD ATG^ embryos revealed mild pericardial effusion without yolk sac edema and a straight tail. A similar effect of the knockdown was detected in both the HPS4^KD e8i8^ and HPS4^KD ATG^ groups which showed no pericardial effusion, yolk sac edema while a straight tail occurred. The HPS5^KD e12i12^ led to the most severe pericardial effusion, yolk sac edema and tail curvature, and the HPS5^KD ATG^ caused a milder degree of pericardial effusion, yolk sac edema but a tail curvature, while CTRL-MO injected larvae showed no signs of an abnormal phenotype. Scale bar = 500 µm. (**B**) Phenotypic changes, indicating glomerular filtration barrier impairment, were determined by grading each larva at 96 hpf in terms of severity of pericardial effusion and yolk sac edema. Applying this grading system, a physiological appearance was scored as a P1 while the most severe edema and pericardial effusion was scored as P4. The number of dead larvae at 96 hpf is also stated. Both HPS1^KD i6e7^ and HPS1^KD ATG^ resulted in either death or aberrance from the normal P1 condition in more than 50%, while a P2 phenotype in HPS1^KD i6e7^ and the P1 was in HPS5^KD ATG^ were the most prominent. HPS3^KD e7i7^ did not show phenotypic changes in comparison with the control group, while the HPS3^KD ATG^ showed a higher death rate but only mild to severe aberration in a minority of the larvae in comparison with the control group. In both HPS3^KD e7i7^ and HPS3^KD ATG^ larvae, a P1 phenotype was the most common. The HPS4^KD e8i8^ induced a higher death rate but only mild aberration in some larvae from the normal phenotype in comparison with the control group. The HPS4^KD ATG^ showed a similar death rate compared to the control while only the minority of larvae displayed a varying degree of aberration. HPS5^KD e12i12^ resulted in either death or deviated from the normal P1 condition in more than 50% of the cases, the P3 was in HPS5^KD e12i12^ the most prominent phenotype. The HPS5^KD ATG^ showed overall similar results as the control group. (**C**) Quantification of maximum fluorescence intensity in the retinal vessel plexus of a *Tg(l-fabp:eGFP-DBP)* transgene zebrafish line depicted in arbitrary units (*AU*). Scatterplot presenting maximum fluorescence intensity of the fish eye was analyzed with ImageJ. All HPS^KD^ groups were compared with the CTRL-MO injected group. HPS1^KD i6e7^, HPS1^KD ATG^, HPS5^KD e12i12^ and HPS5^KD ATG^ led to a decrease of fluorescence intensity, while HPS3^KD e7i7^, HPS3^KD ATG^, HPS4^KD e8i8^ and HPS4^KD ATG^ did not induce a reduction of fluorescence intensity. At least 26 animals were measured for each condition. The error bars indicate the mean ± SEM. n.s. = p ≥ 0.05, ***p ≤ 0.001, ****p ≤ 0.0001 by one-way ANOVA followed by Tukey’s multiple comparisons test.

Pericardial effusion and yolk sac edema can be indicators of circulatory protein loss prevalent in podocytopathies. Induction of an HPS knockdown was carried out via splice or ATG-MO injection in the 1–2 cell stage embryo. In order to determine phenotype changes indicative of renal protein loss, we evaluated each larva at 96 hpf regarding pericardial effusion and yolk sac edema. Depending on the severity of edema and pericardial effusion, a physiological appearance was scored as a P1 while most severe edema and pericardial effusion was scored as P4. Also, the amount of dead fish at 96 hpf was determined. Both HPS1^KD i6e7^ and HPS1^KD ATG^ resulted in either death or deviated from the normal P1 condition in more than 50% (Fig. 4A,B). HPS3^KD e7i7^ did not induce phenotypic changes in comparison with the control group, while the HPS3^KD ATG^ showed a higher death rate but only mild aberration in some larvae from the normal phenotype in comparison with the control group (Fig. 4A,B). HPS4^KD e8i8^ caused a higher death rate compared to the control, however, only a minority deviated from a P1 phenotype (Fig. 4A,B). The majority of HPS4^KD ATG^ larvae displayed a P1 phenotype, while the death rate appeared to be highly similar to the control (Fig. 4A,B). The induction of the HPS5^KD e12i12^ in larvae caused a severe P3 phenotype in most cases, while also the death rate was higher in comparison with the control group (Fig. 4A,B). In contrast, HPS5^KD ATG^ lead to a similar phenotype and death rate as the control MO injection (Fig. 4B). Further information on the death rate and time of death can be obtained from Fig. S3. Interestingly, none of the HPS^KD^ disturbed the glomerular fusion of the pronephros which was analyzed using the *Tg(wt1b:eGFP)* zebrafish line (Fig. S1C). In order to asses protein loss from the circulation, we determined the maximum fluorescence intensity (MFI) in the retinal vessel plexus of *Tg(l-fabp:eGFP-DBP)*. A reduced MFI indicating protein loss was detected in HPS1^KD i6e7^ and HPS5^KD e12i12^ larvae using splice MOs, while HPS3^KD e7i7^ and HPS4^KD e8i8^ did not cause a reduced fluorescence intensity (Fig. 4C). The HPS^KD^ induced by ATG MO injection resembled the effects caused by the splice interfering MO showing a reduction of fluorescence intensity in HPS1^KD ATG^ and HPS5^KD ATG^ in contrast to HPS3^KD ATG^ and HPS4^KD ATG^ that did not result in circulatory protein loss (Fig. 4C).

**Figure 5:**
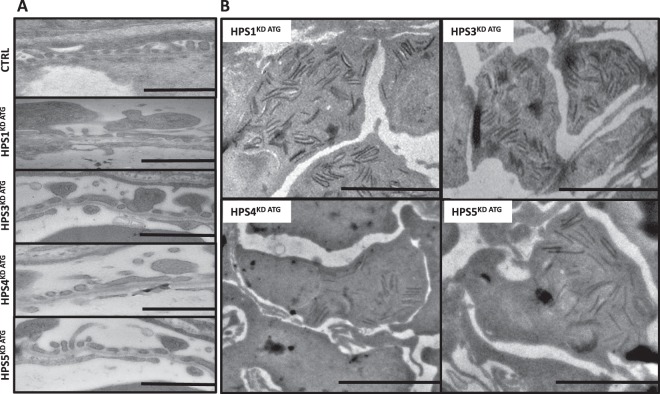
HPS1^KD ATG^, HPS3^KD ATG^, HPS4^KD ATG^ and HPS5^KD ATG^ induce varying degrees of glomerular damage including podocyte effacement and deposition of intracellular debris (zebra bodies). Transmission electron microscopy imaging was used to detect ultrastructural changes in glomeruli after HPS protein knockdown induced by MO injection in *Tg(l-fabp:eGFP-DBP)* transgene zebrafish embryos at a one to four cell stage. Collection of larvae for imaging was done at 120 hpf. (**A**) HPS1^KD ATG^ causes loss of glomerular endothelial fenestration and podocyte effacement. HPS3^KD ATG^ induces mild podocyte effacement. HPS4^KD ATG^ causes podocyte effacement without endothelial injury, while HPS5^KD ATG^ leads to reduced endothelial fenestration and podocyte effacement. Zebrafish injected with a control morpholino (CTRL-MO) show no sign of glomerular damage. Scale bar = 2 µm. (**B**) Induction of HPS protein knockdown of HPS1, -3, -4 and -5 leads to the deposition of intracellular debris primarily in podocytes. Scale bar = 500 nm.

To assess if the observed edema, pericardial effusion and protein loss after HPS^KD^ develop because of structural glomerular defects, we analyzed the cellular components of the fish glomeruli by means of Transmission Electron Microscopy (TEM). Consistent with our phenotypic observations, podocyte effacement is present after knockdown of the different HPS proteins and was particularly prominent after HPS1^KD ATG^ (Fig. 5A). Additionally, a reduction or loss of glomerular endothelial cell fenestration was seen in HPS1^KD ATG^ and HPS5^KD ATG^ zebrafish larvae (Fig. 5A).

Intracellular debris presenting as potential zebra bodies, indicative of a lysosomal associated disorder^13^, were mainly seen in podocytes after the knockdown of any of the HPS proteins (Fig. 5B) Interestingly, the presence of this intracellular debris in podocytes was not limited to zebrafish larvae with phenotype changes associated with proteinuric kidney disease such as edema and pericardial effusion, which suggests that podocytes are associated with the renal phenotype in HPS to a varying degree between the HPS subtypes.

### qPCR determines knockdown efficiency using HPS splice interfering MOs, while knockdown of HPS proteins using ATG blocking MOs in zebrafish larvae does not lead to compensatory expression regulation of other HPS proteins

To evaluate knockdown efficiency of the splice interfering MOs in zebrafish larvae at 72–96 hpf, we used qPCR to determine the fold change of HPS mRNA expression in comparison with HPS expression of the control MO injected group. As shown in Fig. S1A, HPS1^KD i6e7^ efficiency was 30%, while HPS3^KD e7i7^ efficiency was determined at 33%. HPS4^KD e8i8^ efficiency was detected at 32%, HPS5^KD e12i12,^ however, showed the highest efficiency with 81%.

Given that the studied human HPS proteins function primarily by forming BLOCs as heterodimers and heterotrimers, it could be expected that a reduction in the protein expression of one of the HPS genes would lead to a regulatory modification in the expression of the other HPS as a compensatory mechanism. Therefore, by inducing a knockdown of HPS1, -3, -4 and -5 through ATG MO injection in zebrafish larvae, we aimed to detect changes in mRNA expression levels of other HPS proteins using qPCR. The induction of HPS1^KD ATG^ did not cause any RNA expression changes of the other HPS proteins, also HPS4^KD ATG^ did not exert any regulatory effects on the RNA expression of HPS1, despite the reported interaction between HPS1 and HPS4 to form BLOC3 in humans (Fig. S1B)^14^. Although HPS3 and HPS5 are components of the BLOC2 in humans^15^, regulatory RNA expression was neither detected after the induction of HPS3^KD ATG^ nor HPS5^KD ATG^ (Fig. S1B). Interestingly, HPS4 RNA expression appeared to be increased after HPS3^KD ATG^ while no direct interaction within a BLOC has been reported so far in humans, interactions between various BLOCs has been reported previously^16^.

## Discussion

Previous reports of Hermansky-Pudlak Syndrome disease etiology have centered on describing the functional role of the different HPS proteins by developing animal models targeted at the oculocutaneous, pulmonary and hematological aspects of the disease^17,18^. Initial indications of a possible link between certain phenotypic aspects of HPS and progressive renal failure came from the Fawn hooded rat. This model has some characteristics related to HPS, namely hypopigmentation and platelet storage pool deficiencies, but lacks others such as lysosomal accumulation of ceroid lipofuscin^19^. Interestingly, alongside these characteristics, it also harbors an increased genetic predisposition to develop FSGS with proteinuria, subsequently leading to renal failure^20^. None of those studies, however, have focused on the mechanism behind the renal component of HPS. Therefore, our work presents a novel approach that will allow us to address the molecular pathways that lead to renal involvement, in particular to proteinuric kidney disease.

In our model, the induction of HPS1^KD^ using splice interfering and ATG blocking MOs led to the development of classical traits of proteinuric kidney disease in zebrafish. The reduced expression of this protein resulted in pericardial effusion and yolk sac edema, which could be attributed to a defective glomerular filtration barrier (Figs. 4A–C, 5A). Consistent with this, we found a loss of endothelial cell fenestration and podocyte effacement in the glomerulus of the MO-injected fish (Fig. 5A). The fact that HPS1 is actively transcribed in cultured human endothelial cells and podocytes and can be detected in both tubules as well as glomeruli in human kidney sections further highlights the potential involvement of mutations in this protein on the development of a renal phenotype in HPS (Fig. 1A). The first report of an association between a proteinuric kidney disease, FSGS, and Hermansky-Pudlak Syndrome was described in a patient homozygous for a 16 bp duplication in the gene encoding for HPS1^4^. However other co-morbidities present in this case, such as obesity, are also associated with hyperfiltration of the glomerulus, leading to FSGS^21–23^, so a causal relationship between mutations in HPS1 and an increased susceptibility to developing FSGS is difficult to establish from this case. Proteinuric kidney disease associated with HPS-typical traits such as presence of liposfuscin was also described in another patient suffering from HPS, however, prevalent typical IgA-nephropathy histologic findings and crescentic glomerulonephritis prevented the attribution to one specific kidney disease^24^. Taken together, these results highlight the contribution of mutations in and overall downregulation of HPS1 to an increased susceptibility to proteinuric kidney disease within the context of Hermansky-Pudlak Syndrome. The “pale ear” mouse (ep mutation) is a well-established HPS1 mouse model. Previously, lysosomal accumulation of β-galactosidase and β-glucoronidase was described in the “pale ear” mice, while urinary excretion of lysosomal enzymes was shown to be markedly reduced^17,18^.

Although HPS3 expression has been detected predominantly in glomerular cell types and in particular in podocytes (Figs. 1B and 2), a reduced protein expression of HPS3^KD^ induced with splice interfering and ATG blocking MO injection did not lead to severe yolk sac edema and pericardial effusion (Fig. 4A–C) and caused only mild podocyte effacement in TEM imaging (Fig. 5A). Since HPS3^KD ATG^ was associated with lowest survival at 96 hpf despite the at most mildly effected phenotype, it could suggest a regulative role in the development of the zebrafish larvae (Figs. 4B, S3). The present mild podocyte effacement may result from the impaired interaction of HPS3 with clathrin which has been previously shown. This could suggest that HPS3 and therefore the functional unit BLOC2 may be associated with clathrin-mediated endocytosis in podocytes in zebrafish^25,26^.

Reduced expression of HPS4, component of the BLOC3, was associated with a higher death rate in HPS4^KD e8i8^ while only a minority of surviving fish displayed signs of a non-physiologic phenotype (Figs. 4A,B, S3). The HPS4^KD ATG^ was not causative of a death rate exceeding the likelihood of death in comparison with the control group (Figs. 4B, S3). The development of yolk sac edema and pericardial effusion was rare in both HPS4^KD e8i8^ and HPS4^KD ATG^, and proteinuria indicated by fluorescence reduction caused by loss of eGFP-DBP through the GFB was not detected (Fig. 4A–C). The TEM findings after HPS4^KD ATG^ showing podocyte effacement, would suggest proteinuria. The detected expression of HPS4 in humans might be too low to functionally impact the integrity of the GFB (Figs. 2B+5A). A reduced pigment intensity following HPS4^KD ATG^ in zebrafish was present, which further supports the presence of characteristic traits of HPS in this model (Fig. S2B,C).

HPS5, with mRNA expression detected in podocytes (Fig. 1D), belongs to the BLOC2 as HPS3 does. In contrast to the HPS3^KD^, the HPS5^KD e12i12^ and HPS5^KD ATG^ led to the classical traits of proteinuria in zebrafish accompanied with fluorescence reduction caused by eGFP-DBP loss. In accordance with these data, TEM analysis pointed to both podocyte effacement and loss of endothelial cell fenestration in HPS5^KD ATG^. This may account for the relevant phenotypic and functional changes while detected mRNA in both human cell types is fairly low. Daly *et al*. developed an HPS5 knockout model, the snow-white zebrafish, which in their own study mimics completely the HPS5^KD^ induced by MO injection after 4 days post fertilization^27^. In their study, in particular yolk sac edema was detected, which furthermore supports the claim that HPS5^KD^ is associated with circulatory protein loss^27^. Additionally, the phenotypic resemblance between the knockout and knockdown model, including the reduced pigment intensity (Fig. S2B,C), is suggestive of negligible “off-target” hits of the MO.

The induction of HPS knockdowns led to the striking finding of the accumulation of intracellular debris resembling the previously described zebra bodies in lysosomal disorders (Fig. 5B)^13^. The detection of this accumulation serves as a proof of principle of lysosome dysfunction which shows the effectiveness and specificity of the MO-induced reduction of HPS protein expression.

To conclude, to our knowledge, we are the first to investigate the association of HPS with proteinuric kidney disease, while multiple studies and reports have shown that the relevance of renal disease has been underestimated.

## Materials and Methods

### Cell culture

Culture conditions of conditionally immortalized human podocytes have been described previously^28^. Under permissive conditions at 33 °C, podocytes proliferated. Cultivation at 37 °C led to inactivation of the SV40 T-antigen to induce cell differentiation. Culture medium for human podocytes was RPMI 1640 Medium (Roth, Karlsruhe, Germany) with 10% fetal calf serum (FCS; PAA Laboratories, Pasching, Australia), 1% Penicillin/Streptomycin and 0.1% human insulin (Sigma-Aldrich, St. Louis, MO). Human glomerular mesangial cells (Cell systems, Kirkland, WA) proliferated at 37 °C in RPMI 1640 Medium (Roth, Karlsruhe, Germany) with 10% fetal calf serum (FCS; PAA Laboratories, Pasching, Australia), 1% Penicillin/Streptomycin and 0.1% human insulin (Sigma-Aldrich, St. Louis, MO). Human glomerular microvasculature endothelial cells (Cell systems, Kirkland, WA) were cultivated on CSC complete media (Cell systems, Kirkland, WA). To the medium, Bac-Off® (antibiotic) and CultureBoost-R™ (human recombinant growth factors; Cell systems, Kirkland, WA) were added, culture conditions were 37 °C and 5% CO_2_.

### qPCR

Total mRNA was purified either from cultured human podocytes, mesangial cells, and endothelial cells or from larval zebrafish at 72, 96 or 120 hpf using the RNAeasy Minikit (Qiagen, Hilden, Germany) according to the manufacturer’s protocol. Reverse transcription of 1 µg RNA, was done using Oligo(dT)primer (Promega, Madison, WI, USA), and Random primers (Promega, Madison, WI, USA) that were incubated at 70 °C for 10 min followed by retrotranscription using M-MLV RT buffer (Promega, Madison, WI, USA), with dNTPs (Roche, Mannheim, Germany), and M-MLV reverse transcriptase (Promega, Madison, WI, USA) at 42 °C for 90 min and at 70 °C for 10 min. Sybr green-based qPCR was performed using the TaKaRa SYBR Premix Ex Taq™ II (TaKaRa Bio USA, Mountain View, CA, USA).

### Immunohistochemistry

Data were acquired using the Human Protein Atlas version 18 (https://www.proteinatlas.org/)^29^. Antibodies that were used for immunohistochemistry are available in the supplementary section (Table S4).

### *In vivo* studies in zebrafish

Zebrafish (*Danio rerio*) were raised and mated at 28.5 °C. Zebrafish were kept and handled in standard E3 solution, as previously described^30^. The Nanoject II injection device (Drummond Scientific, Broomall, PA) was used to inject 4.6 nL of MO diluted 1:1 with injection buffer (20 mmol/L HEPES, 200 mmol/L KCl, and 0.75% phenol red) into one- to two-cell stage fertilized embryos as previously described^9,11^. Morpholinos were injected at the following concentrations: HPS1^KD i6e7^ (100 µM), HPS1^KD ATG^ (100 µM), HPS3^KD e7i7^ (50 µM), HPS3^KD ATG^ (25 µM), HPS4^KD e8i8^ (250 µM), HPS4^KD ATG^ (100 µM), HPS5^KD e12i12^ (250 µM) and HPS5^KD ATG^ (100 µM) as well as MO-CTRL (100–250 µM). All morpholino sequences were BLASTed using ensembl to check for potential off-target hits. The strategy for screening off-target hits was as follows: First only hits falling within a named gene were examined. Second if the potential off-target hit gene and MO fragment binding orientations were not compatible for binding the RNA the hit was excluded. Third, the binding position of remaining hits was scrutinized for its potential to interfere with translation or splicing in a manner similar to a morpholino. Potential translation blocking was excluded unless binding of the MO fragment occurred in an identified 5’UTR or within 25nt downstream of the start codon. Splice blocking potential was excluded unless a fragment bound across a splice junction, within 5nt from an end of an intron or 2nt from the end of an exon. Lastly confirmed hits with an RNA-morpholino binding Tm < 70 °C as determined by a Genetools algorithm could also be excluded. MOs were only purchased from Gene Tools (Philomath, OR) whenever the aforementioned criteria were met (sequences and off-target hit screening shown in Supplementary Tables S4 and S5). To determine glomerular filtration barrier integrity, we used the transgenic zebrafish-line *Tg(l-fabp:eGFP-DBP)* which expresses a GFP-tagged vitamin D binding protein, as previously described^9,11^. The measurement of the maximum fluorescence intensity of gray-scale images of the retinal vessels of the zebrafish was done with ImageJ (Version 1.60, National Institutes of Health, Bethesda, MD) and reported in arbitrary units (*AU*). To detect developmental defects in the kidney development of HPS^KD^ larvae, we used the *Tg(wt1b:eGFP)* transgenic zebrafish line to visualize the pronephros. The MDI Biological Laboratory (Bar Harbor, ME) animal care committee approved the animal protocol (IACUC protocol #1703).

### Transmission electron microscopy

Larval zebrafish injected with MOs and MO-CTRL were fixed at 120 hpf in 1.5% glutaraldehyde/1% paraformaldehyde, 70 mmol/L NaH_2_PO_4_, and 3% sucrose (pH 7.2). Further preparation of the zebrafish embryos for TEM was performed as stated in previously published protocols^31^. Briefly, the larvae were washed three times in 0.2 mol/L cacodylate buffer and then post fixed in 1% osmium tetroxide for 1 h at room temperature. The specimens were rinsed with cacodylate buffer, dehydrated in a graded ethanol series, infiltrated and then embedded with Epon (Hard Plus Resin 812; Electron Microscopy Sciences, Hatfield, PA) according to the manufacturer’s protocol. Thin-sections of 0.5 and 1 mm were acquired with a Leica RM2165 rotary microtome followed by staining with 0.5% toluidine blue in a 1% sodium tetraborate solution. Identification of the pronephros was performed after toluidine blue staining, semithin (300 nm) and ultrathin (90 nm) sectioning was performed with a Leica Microtome (Leica Microsystems Inc., Buffalo Grove, IL) and transferred onto copper slit grids (Electron Microscopy Sciences, Hatfield,PA). Grids were stained with uranyl acetate (2%) for 30 minutes and lead citrate for 15 minutes with 3 washing steps in between followed by photograph acquisition and evaluation.

### Statistics

The statistical analysis of data was performed using GraphPad Prism 8.1 software. Error bars correspond to SEM. We used one-way ANOVA followed by Tukey’s multiple comparisons test or student’s t test to compare the results of each single test group. A P value of ≤ 0.05 was considered statistically significant. *p ≤ 0.05, **p ≤ 0.01, ***p ≤ 0.001, ****p ≤ 0.0001.

## Supplementary information

Supplementary Figures

Supplementary Legends

## Data Availability

The datasets generated and analysed during the current study are available from the corresponding author on reasonable request.
