# Supplementary material for: Characterizing renal involvement in Hermansky-Pudlak Syndrome in a zebrafish model
Source: Sci Rep. 2019 Nov 27;9:17718. doi: 10.1038/s41598-019-54058-5 (PMC6881439; doi:10.1038/s41598-019-54058-5)
Supplement: Supplementary file 1 — Supplementary Figures [file 41598_2019_54058_MOESM1_ESM.pdf]

Characterizing renal involvement in Hermansky-Pudlak Syndrome in a zebrafish model

Schenk H[1,2]*, Müller-Deile J[1,3]*, Schroder P[2], Bolaños-Palmieri P[1,3], Beverly-Staggs L[2], White R[2], Bräsen JH[4], Haller H[1,2], Schiffer M.[1,3]

[1]Department of Medicine/Nephrology, Hannover Medical School, 30625 Hannover, Germany
[2]Mount Desert Island Biological Laboratory, Salisbury Cove, ME 04672, USA
[3]Department of Nephrology and Hypertension, University of Erlangen-Nurnberg, Erlangen, Germany
[4]Institute of Pathology, Nephropathology Unit, Hannover Medical School, Hannover, Germany

*equal contribution

Supplementary Information

S1

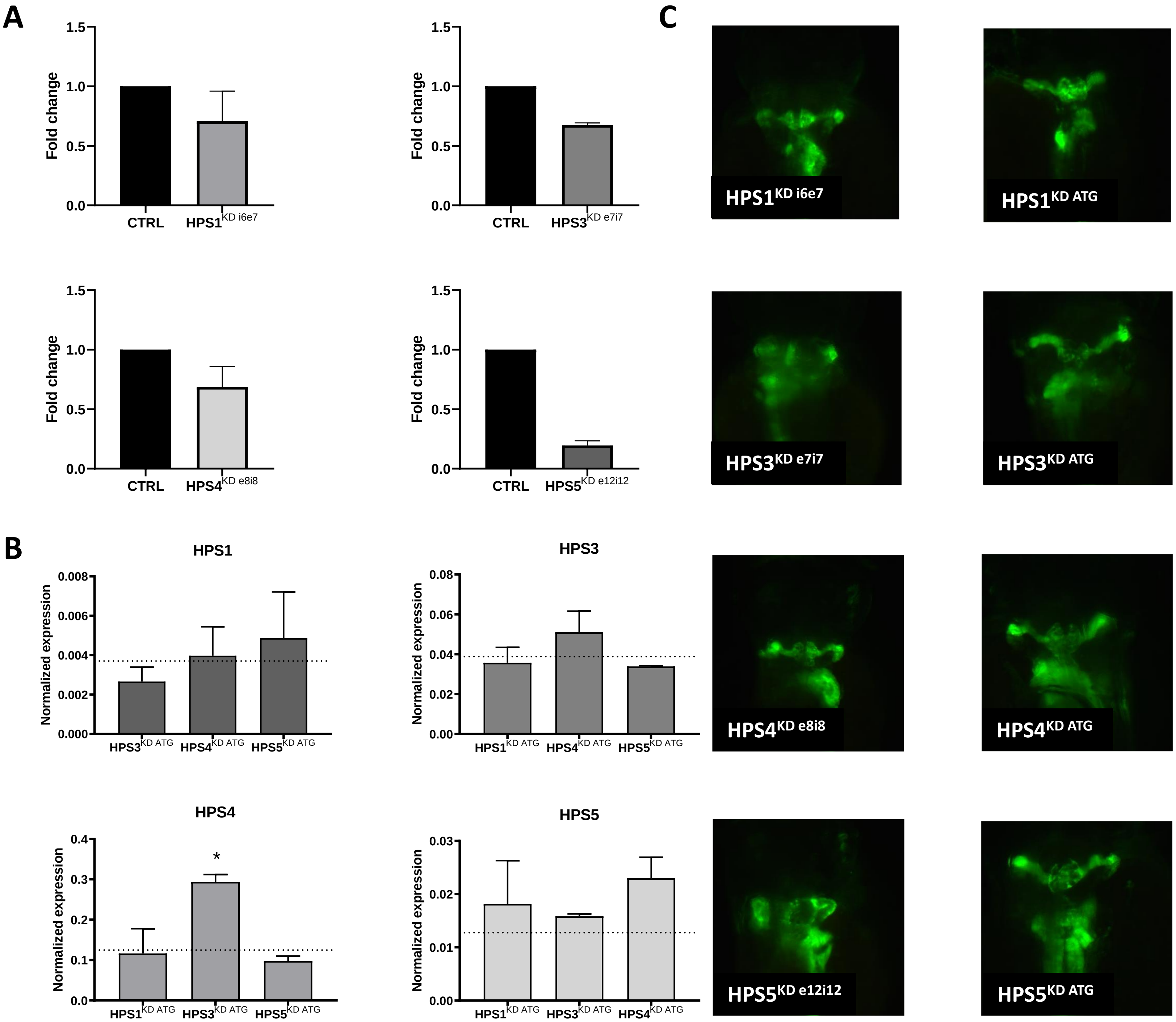
A
Fold change
1.5
1.0
0.5
0.0
CTRL
HPS1KD i6e7
HPS3KD e7i7
HPS4KD e8i8
HPS5KD e12i12
B
HPS1
HPS3
HPS4
HPS5
Normalized expression
HPS1KD ATG
HPS3KD ATG
HPS4KD ATG
HPS5KD ATG
*
C
HPS1KD i6e7
HPS1KD ATG
HPS3KD e7i7
HPS3KD ATG
HPS4KD e8i8
HPS4KD ATG
HPS5KD e12i12
HPS5KD ATG

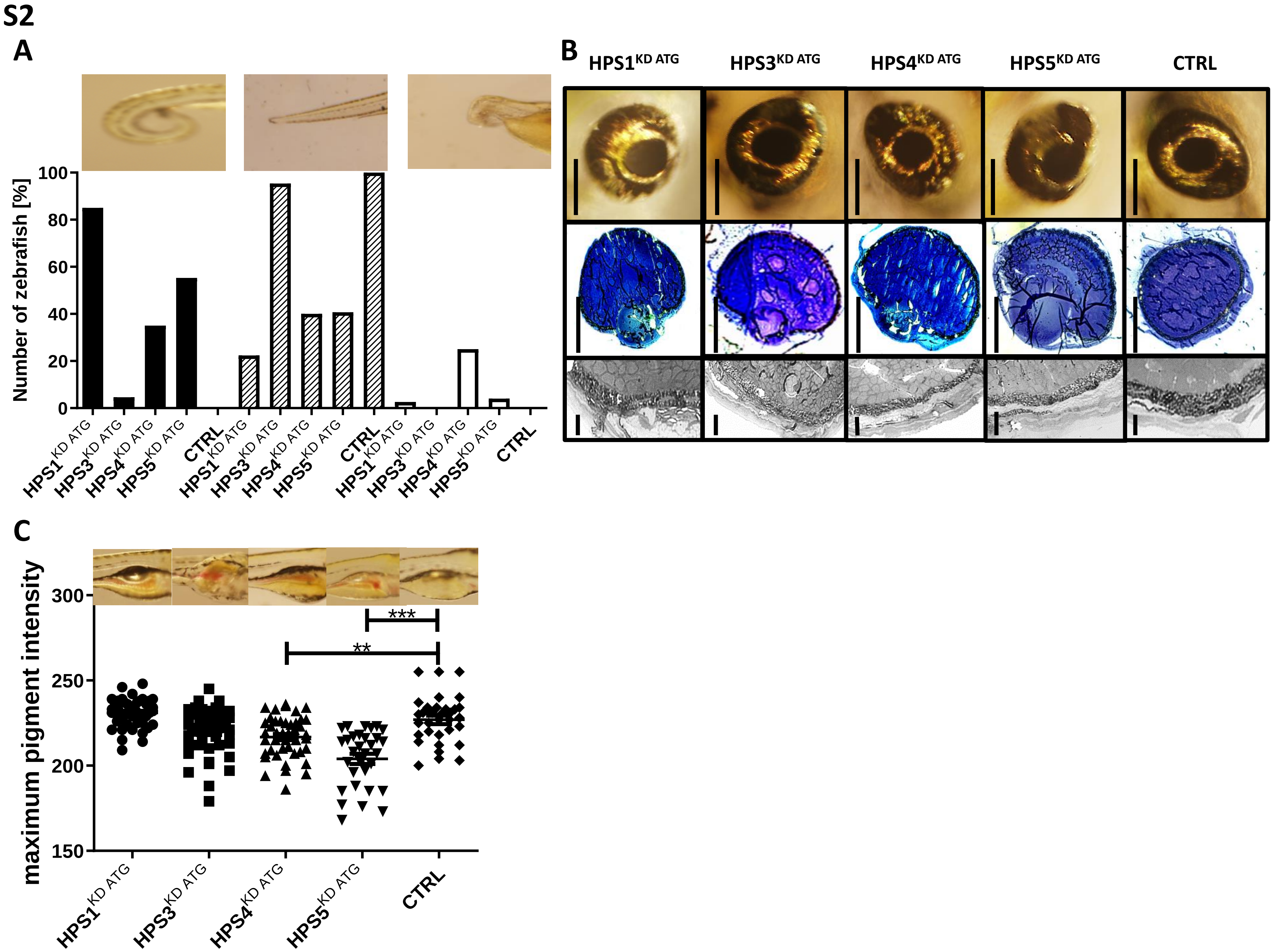

S2
A
Number of zebrafish [%]
100
80
60
40
20
0
HPS1KD ATG
HPS3KD ATG
HPS4KD ATG
HPS5KD ATG
CTRL
B
HPS1KD ATG
HPS3KD ATG
HPS4KD ATG
HPS5KD ATG
CTRL
C
maximum pigment intensity
300
250
200
150
***
**
HPS1KD ATG
HPS3KD ATG
HPS4KD ATG
HPS5KD ATG
CTRL

S3

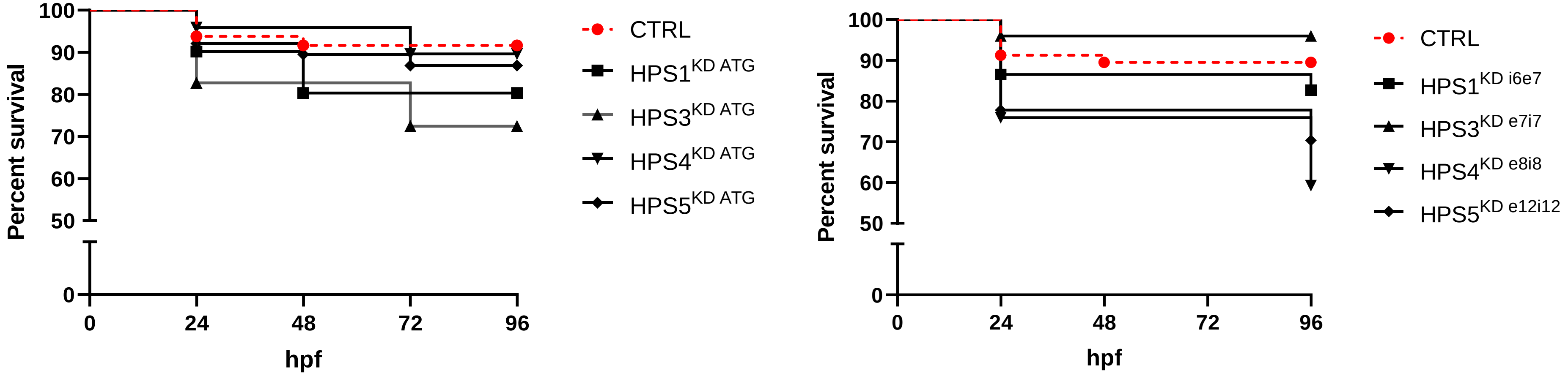
Percent survival
100
90
80
70
60
50
0
0
24
48
72
96
hpf
CTRL
HPS1$^{KD\ ATG}$
HPS3$^{KD\ ATG}$
HPS4$^{KD\ ATG}$
HPS5$^{KD\ ATG}$
Percent survival
100
90
80
70
60
50
0
0
24
48
72
96
hpf
CTRL
HPS1$^{KD\ i6e7}$
HPS3$^{KD\ e7i7}$
HPS4$^{KD\ e8i8}$
HPS5$^{KD\ e12i12}$

S4

Primers human	Sequence
HPS1 forward	GAAGTGAGGACCAGGGATGC
HPS1 reverse	CTCGGTGACTGGCTCATGG
HPS3 forward	ACAGCGAGGCTGGAGATTAT
HPS3 reverse	AGCACGTAGAAATGTAGCTTTG
HPS4 forward	CTCGCCTCACATTCTCGCT
HPS4 reverse	GGGTGCTGACAATCAGTCCT
HPS5 forward	ACTGCTGTTAAAAAGGTGAGGA
HPS5 reverse	GCCATTTAGCCAGAAAGCTGA
HPRT forward	TGACACTGGCAAAACAATGCA
HPRT reverse	GGTCCTTTTCACCAGCAAGCT

Antibodies human	Stock number
HPS1	HPA061260
HPS3	HPA046281
HPS4	CAB033106
HPS5	n.a.

Primers zebrafish	Sequence
HPS1.1 forward	AGTGCTGCTCACCAAGATCC
HPS1.1 reverse	AAGTAGTACACCGACACCGC
HPS3.1 forward	AGACCGTCACACTTACAGCC
HPS3.1 reverse	CATACACCTGCAGCATCTCGG
HPS4.1 forward	GCCCTCTGAGCTGAACATGA
HPS4.1 reverse	TGGATTGCAGTGAGACCTGC
HPS5.1 forward	TGCTGGAGATATGGGTGGGA
HPS5.1 reverse	CTGAGCCCTTGCCGAGTTTA
HPRT forward	ACCAAAACACTATGCGGCTG
HPRT reverse	GTGTCCACCCATGTCCTTCA
HPS1.2 forward	TAACAGCAGCATGGACCGAG
HPS1.2 reverse	TTGCTGGCATTACGACTGGA
HPS3.2 forward	ACGTCATCACCAGGAACGC
HPS3.2 reverse	CTGAACCCGAGCTGCTACTG
HPS4.2 forward	TACAGCCAGGATAACCGTGTG
HPS4.2 reverse	CCCACAGATGAGCTCAGACC
HPS5.2 forward	AGGTGGATAACGACTCTGCG
HPS5.2 reverse	AGAAACTGCAAGCCGAGGTC

Morpholino zebrafish	Sequence
HPS1 ATG	5'AGTGTCATTGTTTCTGAATCCTGGT3'
HPS3 ATG	5'TGTGTGCTGAGCTGCGTTACCTTCA3'
HPS4 ATG	5'CGGCCAAAACAGCACTTTCAGCCAT3'
HPS5 ATG	5'ACTCCACAGGAACCACAGGAATCAT3'
CTRL	5'CCTCTTACCTCAGTTACAATTTATA3'
HPS1 i6e7	5'TGCCTGAATGGAACCAGAGAACTCT3'
HPS3 e7i7	5'TGTGTGCTGAGCTGCGTTACCTTCA3'
HPS4 e8i8	5'TTATGAATGTGATTTGCACCTGGCT3'
HPS5 e12i12	5'TTGTAAGTCTCTCTCAGTCTCACCG3'

S5

Morpholino			Number of potential off-targets							
**Name**	**Sequence**	**Complementary reverse sequence**	**With binding greater than 14bp**	**Unknown overlapping gene**	**With wrong orientation to the gene**	**Within intron region**	**Within exon region**	**Within 3'UTR region**	**Within 5'UTR region**	**Within exon-intron boundary**
hps1 5' UTR	AGTGTCATT GTTTCTGAAT CCTGGT	ACCAGGATTC AGAAACAATG ACACT	253	90	75	86	-	-	si:dkey-118k5.3 and igf1rb	-
hps1 i6e7	TGCCTGAAT GGAACCAGA GAACTCT	AGAGTTCTCTG GTTCCATTCAG GCA	22	11	5	5	1	-	-	-
hps3 ATG	AGTTGTAAA CGTGGACCA TTTCTAC	GTAGAAATGG TCCACGTTTAC AACT	5	2	2	1	-	-	-	-
hps3 e7i7	TGTGTGCTG AGCTGCGTT ACCTTCA	TGAAGGTAAC GCAGCTCAGC ACACA	10	5	2	2	-	dstyk	-	-
hps4 ATG	CGGCCAAAA CAGCACTTTC AGCCAT	ATGGCTGAAA GTGCTGTTTTG GCCG	359	137	86	40	4	sybl1	-	-
hps4 e8i8	TTATGAATGT GATTTGCAC CTGGCT	AGCCAGGTGC AAATCACATT CATAA	59	30	17	12	-	-	-	-
hps5 ATG	ACTCCACAG GAACCACAG GAATCAT	ATGATTCCTGT GGTTCCTGTGG AGT	151	39	32	59	20	-	-	exon junction in 5'UTR of sybl1
hps5 e12i12	TTGTAAGTCT CTCTCAGTCT CACCG	CGGTGAGACT GAGAGAGACT TACAA	229	102	78	46	3	-	-	-
