# Supplementary material for: Characterizing renal involvement in Hermansky-Pudlak Syndrome in a zebrafish model
Source: Sci Rep. 2019 Nov 27;9:17718. doi: 10.1038/s41598-019-54058-5 (PMC6881439; doi:10.1038/s41598-019-54058-5)
Supplement: Supplementary file 2 — Supplementary Legends [file 41598_2019_54058_MOESM2_ESM.docx]

**Characterizing renal involvement in Hermansky-Pudlak Syndrome in a zebrafish model**

Schenk H^1,2^*, Müller-Deile J^1,3^*, Schroder P^2^, Bolaños-Palmieri P^1,3^, Beverly-Staggs L^2^, White R^2^, Bräsen JH^4^, Haller H^1,2^, Schiffer M.^1,3^

^1^Department of Medicine/Nephrology, Hannover Medical School, 30625 Hannover, Germany
^2^Mount Desert Island Biological Laboratory, Salisbury Cove, ME 04672, USA
^3^Department of Nephrology and Hypertension, University of Erlangen-Nurnberg, Erlangen, Germany
^4^Institute of Pathology, Nephropathology Unit, Hannover Medical School, Hannover, Germany

*equal contribution

**Supplementary Legends**

Fig. S1

**Knockdown efficiency using qPCR following HPS^KD^ and expression of mRNA of HPS subtypes in *Tg(l-fabp:eGFP-DBP)* transgene zebrafish line** **after HPS^KD^.** (A) Knockdown efficiency of the splice MO in zebrafish larvae at 72-96 hpf was measured using qPCR to detect the fold change of HPS mRNA expression in comparison with HPS expression of the control MO injected group. The lowest efficiency was for the HPS1^KD i6e7^ with 30 %, while the highest was HPS5^KD^ ^e12i12^ with 81 %. (B) qPCR reveals mRNA levels of HPS subtypes after MO injections, mRNA levels are depicted as a ratio relative to the control-MO injection. mRNA expression of HPS subtypes is normalized to the housekeeping gene HPRT. HPS1 mRNA level after induction of HPS3^KD ATG^, HPS4^KD^ ^ATG^ and HPS5^KD^ ^ATG^ is shown. HPS3 mRNA level after induction of HPS1^KD ATG^, HPS4^KD^ ^ATG^ and HPS5^KD^ ^ATG^ is depicted. HPS4 mRNA level after induction of HPS1^KD ATG^, HPS3^KD ATG^ and HPS5^KD^ ^ATG^ is shown. HPS5 mRNA level after induction of HPS1^KD ATG^, HPS3^KD ATG^ and HPS4^KD^ ^ATG^ is shown. Error bars correspond to SEM between three independent experiments (n = 3), *p≤0.05 by one-way ANOVA followed by Tukey's multiple comparisons test.

Fig. S2

**Phenotype evaluation of HPS1^KD ATG^, HPS3^KD ATG^, HPS4^KD^ ^ATG^, HPS5^KD^ ^ATG^ as well as MO-CTRL reveals that reduced HPS4 and HPS5 expression decreases pigment intensity of zebrafish larvae.** (A) Categorization of larval tail phenotype compared to CTRL-MO injected larvae 96 hpf. The presence of a curved tail was detected predominately HPS1^KD ATG^ larvae, while HPS3^KD ATG^ caused a minor increase in curved tails. HPS4^KD ATG^ and HPS5^KD ATG^ led to an increase in the development of a curved tail. (B) Display of the ocular pigmentation in HPS1^KD ATG^, -3^KD ATG^, -4^KD ATG^ and -5^KD ATG^ zebrafish compared to CTRL-MO injected larvae at 96 hpf. While HPS1^KD ATG^ fish showed a comparable pigmentation in comparison with the CTRL-MO injected larvae, HPS3^KD ATG^, -4^KD ATG^, and -5^KD ATG^ larvae showed ocular hypopigmentation. (C) Scatterplot presenting maximum pigment intensity of the fish cutis was analyzed with ImageJ. Quantification of the cutaneous pigmentation in HPS1^KD ATG^, -3^KD ATG^, -4^KD ATG^ and -5^KD ATG^ zebrafish compared to CTRL-MO injected larvae at 96 hpf depicted in arbitrary units (*AU*). HPS4^KD ATG^ and HPS5^KD ATG^ injected fish had a reduced maximum pigment intensity compared with the CTRL-MO injected group, HPS1^KD ATG^ and HPS3^KD ATG^ showed no hypopigmentation compared to the CTRL-MO injected group.

Fig. S3

**Survival rate of HPS1^KD i6e7^, HPS3^KD e7i7^, HPS4^KD^ ^e8i8^, HPS5^KD^ ^e12i12^ and HPS1^KD ATG^, HPS3^KD ATG^, HPS4^KD^ ^ATG^, HPS5^KD^ ^ATG^ at the timepoints 24, 48, 72 and 96 hpf.** While both HPS1^KD i6e7^ and HPS1^KD ATG^ showed a similar survival, HPS3^KD ATG^ was associated with a lower survival percentage compared to the HPS3^KD e7i7^. HPS4^KD^ ^ATG^ however showed a higher survival rate compared to the HPS4^KD^ ^e8i8^. HPS5^KD^ ^ATG^ had a higher survival rate compared to HPS5^KD^ ^e12i12^.

Table S4

The primer pairs used to detect expression levels of the HPS mRNA and the housekeeping gene HPRT in human and zebrafish are stated. The antibodies to detect renal expression of HPS proteins are furthermore stated as used in the Human Protein Atlas version 18 (https://www.proteinatlas.org/). The used morpholino (MO) sequences to target HPS subtypes as well as the control morpholino (CTRL-MO) are stated.

Table S5

Off-target hit screen of the morpholinos used to knockdown the HPS proteins.
